# Role of glia and extracellular matrix in controlling neuroplasticity in the central nervous system

**DOI:** 10.1007/s00281-023-00989-1

**Published:** 2023-04-13

**Authors:** Egor Dzyubenko, Dirk M. Hermann

**Affiliations:** Department of Neurology and Center for Translational Neuro- and Behavioral Sciences (C-TNBS), University Hospital Essen, Hufelandstr. 55, 45147 Essen, Germany

**Keywords:** Neuroplasticity, Astrocyte, Microglia, Neuron-glia interactions, Inflammation, Extracellular matrix

## Abstract

Neuronal plasticity is critical for the maintenance and modulation of brain activity. Emerging evidence indicates that glial cells actively shape neuroplasticity, allowing for highly flexible regulation of synaptic transmission, neuronal excitability, and network synchronization. Astrocytes regulate synaptogenesis, stabilize synaptic connectivity, and preserve the balance between excitation and inhibition in neuronal networks. Microglia, the brain-resident immune cells, continuously monitor and sculpt synapses, allowing for the remodeling of brain circuits. Glia-mediated neuroplasticity is driven by neuronal activity, controlled by a plethora of feedback signaling mechanisms and crucially involves extracellular matrix remodeling in the central nervous system. This review summarizes the key findings considering neurotransmission regulation and metabolic support by astrocyte-neuronal networks, and synaptic remodeling mediated by microglia. Novel data indicate that astrocytes and microglia are pivotal for controlling brain function, indicating the necessity to rethink neurocentric neuroplasticity views.

## Introduction

Neuroplasticity is a key to understanding brain development, learning, and homeostatic regulation in the central nervous system (CNS). In a broad sense, the term “neuroplasticity” refers to the ability of nervous tissue to change during normal functioning or in pathology. The mechanisms of neuronal plasticity include modulation of synaptic strength (i.e., synaptic plasticity), structural remodeling, and adjustment of intrinsic neuronal properties such as excitability or firing rate. Although neuroplasticity is traditionally associated with neuron-based pathways, recent experimental data emphasize the role of regulatory mechanisms involving glial cells and the brain extracellular matrix (ECM). In the mature brain, neurogenesis and axonal sprouting are inhibited by the ECM [1], but gliogenesis remains active [2]. Ample evidence indicates that glia (meaning “glue” in Greek) are vividly interacting brain cells that communicate via gap junctions and cytokines in health and disease [3]. The four major glial cell populations in the CNS are NG2-glia, oligodendrocytes, astrocytes, and microglia. NG2-glia is essential for the renewal of glial cells (for review, see [4]), and oligodendrocytes are required for myelin formation (for review, see [5]). Neurons and glial cells collectively shape the brain ECM [6], which is an important mediator of intercellular signaling in the extracellular space [7]. ECM is mainly composed of polysaccharides and proteoglycans that act as extracellular scaffolds and provide a highly regulated environment for intercellular communication by regulating the diffusion of metabolites and signaling molecules [8]. ECM components regulate the hydrodynamics of the extracellular space, compartmentalize cell surfaces, and bind signaling mediators. The conventional classification of ECM in the brain parenchyma includes synaptic, interstitial, and condensed matrices of perineuronal nets [9, 10]. The different neuroplasticity-regulating properties of these matrices may vary based on their molecular composition and localization. While astrocytes produce a major part of ECM components, microglia remodel the ECM during neuroinflammation and physiological immune surveillance in the brain. In this review, we highlight the critical role of astrocytes, microglia, and ECM in the regulation of neuronal activity and plasticity at different organizational levels—from single synapses to networks.

### Astrocytic cradle of the synapse

**Fig. 1:**
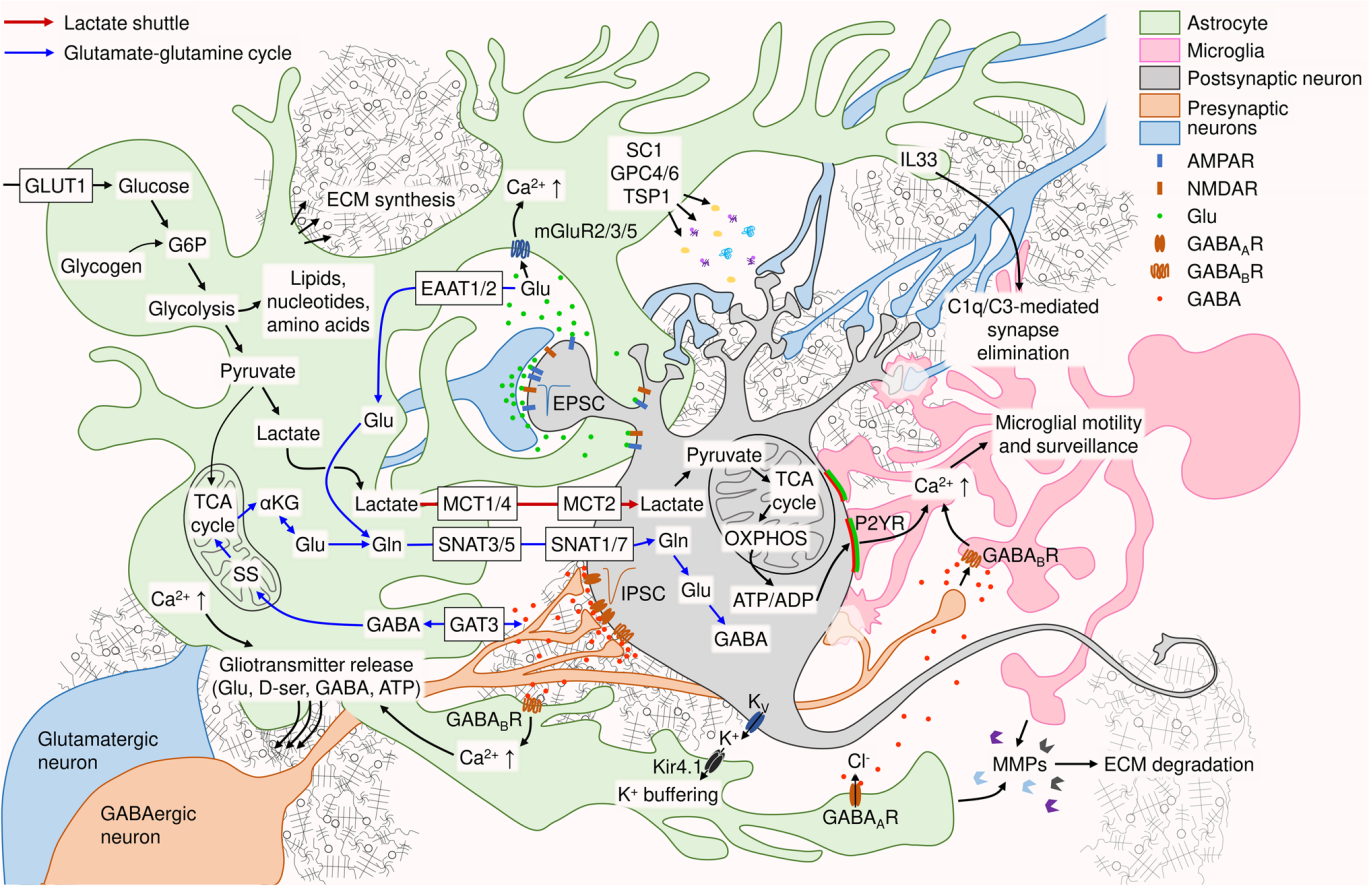
Graphical summary of neuroplasticity mechanisms mediated by glia. Intimate interactions between astrocytes, microglia, and ECM allow for dynamic control of neuronal activity and synaptic transmission. While astrocytes regulate neuronal function by providing metabolic support, modulating neurotransmission, and producing ECM, microglia can sculpt neuronal synapses in an activity-dependent manner. Abbreviations: αKG, α-ketoglutarate; ATP/ADP, adenosine tri- and diphosphate; AMPAR, AMPA receptor; D-ser, D-serine; EAAT, excitatory amino acid transporter; ECM, extracellular matrix; EPSC, excitatory postsynaptic current; IPSC, inhibitory postsynaptic current; G6P, glucose-6-phosphate; GABAAR, ionotropic GABA receptor A; GABABR, metabotropic GABA receptor B; GAT3, GABA transporter 3; Glu, glutamate; Gln, glutamine; GLUT1, glucose transporter 1; IL33, interleukin 33; mGluR, metabotropic glutamate receptor; MMPs, matrix metalloproteases; MCT, monocarboxylate transporter; NMDAR, NMDA receptor; OXPHOS, oxidative phosphorylation; P2YR, purinergic P2Y receptor; SNAT, sodium-coupled neutral amino acid transporter; SS, succinic semialdehyde; TCA cycle, tricarboxylic acid cycle (Krebs cycle)

Astrocytes received their name in the late nineteenth century because of the stellate appearance that was revealed by Camillo Golgi and Santiago Ramón y Cajal using the silver-chromate and gold chloride-sublimate techniques, correspondingly [11]. Today, immunohistochemical labeling of glial acidic fibrillary protein (GFAP), which remains one of the most widely used astrocytic marker proteins, shows similar star-like morphology. However, dye-filling techniques and genetic labeling of astrocytic membrane proteins reveal a dense pattern of highly ramified branches [12]. Therefore, the ground truth is that astrocytes are rather dandelion like than stellate. The thin astrocytic processes enwrap presynaptic terminals and dendritic spines of excitatory synapses [13], creating the spongiform microdomains [14] that provide the structural background for local astrocyte-synapse communication. Emphasizing the intimate proximity between the astrocyte and the synapse, astrocytic perisynaptic processes are called astrocytic cradles [15]. The essential role of astrocytic cradles for controlling synaptic transmission is reflected by the tripartite synapse concept [16] and summarized in Fig. 1.

In tripartite synapses, astrocytes enhance the adhesion between pre- and postsynaptic membranes. While the key synaptic adhesion molecules of the neurexin-neuroligin complex are predominantly synthesized by neurons, astrocytes produce ephrins, integrins, and cadherins, and promote the expression of other cell adhesion molecules of the immunoglobulin superfamily (IgCAMs) including SynCAMs and NCAMs to further stabilize the synapse. The adhesion molecules released by astrocytes promote the formation and stabilize synapses, which makes them essential for maintaining synaptic transmission [17]. Astrocytes also produce glypicans, the heparan sulfate proteoglycans that induce the formation of functional synapses and modulate synaptic plasticity by promoting glutamate receptor clustering [18]. In addition, astrocytes produce other ECM proteins such as thrombospondins and hevin, which have been shown to regulate synapse formation and stability. For a more exhaustive review of cell adhesion in the tripartite synapse, see [19]. The synaptic ECM components produced by astrocytes have long lifetimes [20, 21] and actively modulate synaptic activity [22]. The implications of ECM in synaptic activity regulation have led to the emergence of the more recent tetrapartite synapse concept [23].

On a functional level, astrocytic cradles dynamically control neurotransmitter concentration. Perisynaptic astrocytic membranes create diffusion barriers that limit the spillover of neurotransmitters [24] and express excitatory amino acid transporters (EAATs). EAAT1 (also known as GLAST1 and SLC1A3) and EAAT2 (also known as GLT1 and SLC1A2) mediate rapid reuptake of glutamate, thereby shaping postsynaptic current responses [25]. The expression and uptake capacity of these transporters depend on neuronal activity [26, 27] and are regulated by astrocytic Ca/calmodulin-dependent kinase CaMKII [28, 29]. Interestingly, EAATs 1 and 2 can be synthesized locally in the astrocytic processes [30], which allows for the fast upregulation of glutamate reuptake if necessary.

Inhibitory synapses are predominantly established directly on neuronal somas or dendritic shafts and rarely localize to dendritic spines [31]. To the best of our knowledge, there is no direct evidence of astrocytic cradle formation around inhibitory synapses. However, astrocytes actively sequester GABA from the perisynaptic space via the high-affinity GABA transporter GAT3 [32], providing a reuptake mechanism similar to those for glutamate. Thus, neurotransmitter uptake by astrocytes regulates both excitatory and inhibitory signaling in neuronal networks.

### Gliotransmission and neuronal activity regulation

Astrocytes regulate neuronal activity not only by removing neurotransmitters from the extracellular space but also by releasing them in an activity-dependent manner. To describe this mechanism, the term “gliotransmission” has been coined, and the neurotransmitters released from astrocytes are commonly called gliotransmitters [33]. Astrocytes express membrane receptors for the vast majority of mammalian neurotransmitters [34], and, although they do not generate neuron-like action potentials, astrocytes respond to neurotransmitter application and neuronal stimulation (e.g., after sensory stimuli) with transient elevations of intracellular Ca^2+^ concentrations [35, 36]. Analyzing Ca^2+^ signals, also called Ca^2+^ events, is currently the key method for understanding the physiology of astrocyte-neuronal interactions [37, 38]. Depending on the strength of neuron-to-astrocyte stimulation, Ca^2+^ events may localize to the peripheral astrocytic processes (microdomain activity) or spread over the entire cell and its neighbors (global events or waves). While the microdomain Ca^2+^ activity can be triggered by membrane transporters and ion channels [39], the major Ca^2+^ events are mediated by inositol triphosphate (IP_3_) signaling and store-operated Ca^2+^ release [40]. In the seminal works [41, 42], the Ca^2+^ waves were detected in cultured astrocytes after norepinephrine (NE) application or after prolonged electrical stimulation. More recent in vivo experiments identified the critical importance of astrocytic calcium signaling for neuronal activity regulation [43–45].

Ca^2+^ events trigger the release of gliotransmitters, which regulate synapse activity locally, heterosynaptically, or globally by potentiating or inhibiting synaptic transmission and neuronal excitability (for review, see [33, 46]). Interestingly, astrocytes use similar molecular machinery for the vesicular release of gliotransmitters [47, 48]. In excitatory synapses, the release of astrocytic glutamate and D-serine mediates synaptic potentiation [48–50] and stimulates the barrage firing of inhibitory interneurons [51]. On the other hand, ATP/adenosine release from astrocytes stimulated by both excitatory and inhibitory activity induces heterosynaptic suppression of glutamatergic synapses [43, 52]. In inhibitory synapses, GABA stimulates astrocytic GABA_B_ receptors, induces long-lasting Ca^2+^ oscillations [53], and stimulates excitatory neurotransmission via glutamate release [54]. Conversely, GAT3 transporter activity following GABAergic stimulation induces astrocytic ATP release and inhibitory synapse potentiation [55]. Gliotransmission therefore can act as a jack of all trades in neuronal activity regulation. However, the selection mechanisms determining astrocytic responses to neuronal stimulation remain largely unknown.

**Fig. 2:**
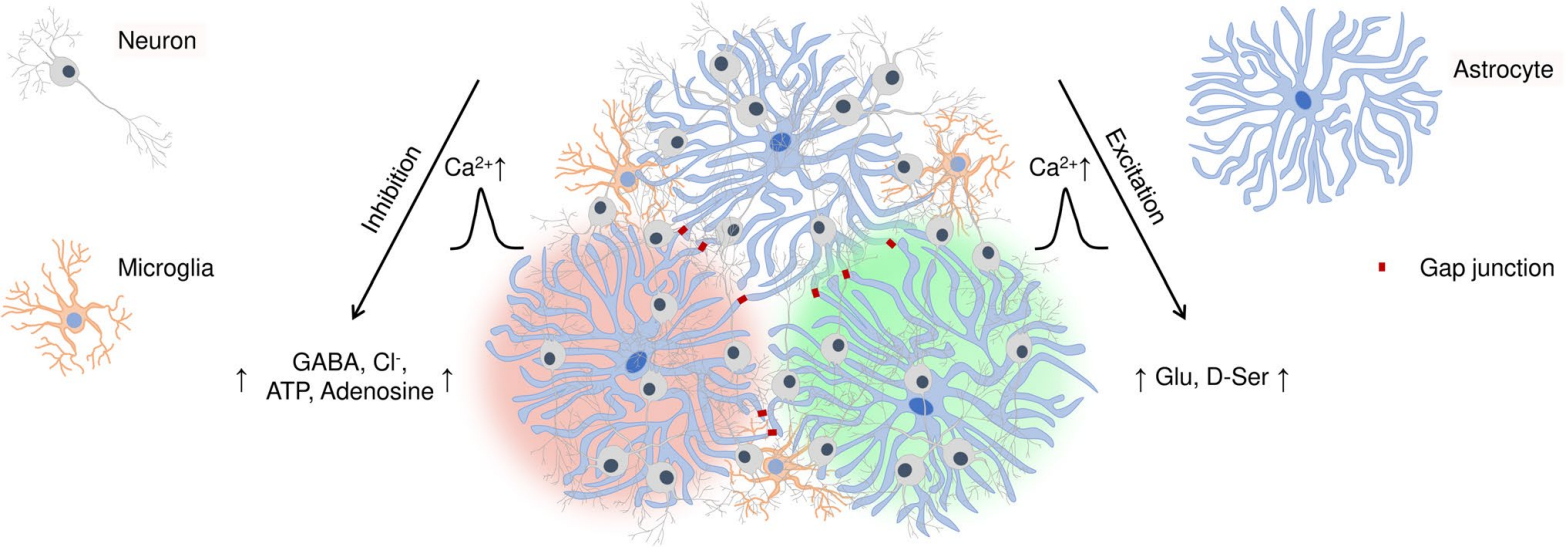
Propagation of excitatory and inhibitory signals in neuron-glial networks. On the mesoscale level of organization, inhibition-excitation balance in local brain networks can be orchestrated by calcium signals propagating through adjacent astrocytes

The described mechanisms of neuron-glia interactions provide flexible modulation of neuroplasticity and indicate a high capability of brain networks for self-regulation. Due to technical difficulties, most experimental studies that we reviewed here were conducted on the sub-cellular and cellular levels, making it yet challenging to conclude how the key neuroplasticity mechanisms mediated by glia integrate on the functional network level. Emerging evidence indicates that astrocytic signaling is essential for neuronal synchronization and inhibitory network refinement [56, 57]. Astrocytes can modulate the efficacy of both excitatory and inhibitory synapses, thereby extending the dynamic range of neuroplasticity and increasing the computational power of local circuits [57] by tuning the excitation-inhibition balance. Recently, it was shown that astrocytes encode spatial information, and the expected reward location can be decoded from their activity in an awake mouse brain [58]. Based on the available data, we propose that multicellular interactions in neuron-glial networks promote ranged propagation of inhibition and excitation and support excitation-inhibition balance in local neuronal networks (Fig. 2).

Although single astrocytes occupy distinct territories and establish non-intersecting islands of synaptic regulation [59], they are functionally connected into a syncytium-like network by gap junctions [60]. Astrocytic coupling via connexin 43 and 30 channels allows for intercellular Ca^2+^ signaling and metabolic coupling, which is necessary for preventing epileptiform activity [61, 62].

Through gap junctions, the traveling Ca^2+^ events may transfer excitatory or inhibitory stimuli from the territory of one astrocyte to another. Thereby, astrocytic networks can regulate neuronal synchronization on a slower time scale than provided by synaptic neurotransmission. Neurotransmitter release from the astrocytes is less area restricted than synaptic release and can therefore affect extrasynaptic receptors of multiple neurons within the astrocytic territory [56], thereby regulating the excitation-inhibition balance without remodeling synaptic connectivity. Tonic inhibition by astrocytic GABA release [63, 64] and the efflux of chloride via astrocytic GABAA channels [65] efficiently modulates network inhibition. Similarly, excitatory gliotransmission and stimulation of extrasynaptic AMPA and NMDA receptors [33, 46] can contribute to spreading excitation.

### Potassium buffering in astrocytes and perineuronal nets

While the control over neurotransmitter concentrations regulates the amplitude, frequency, and dynamics of postsynaptic responses, neuronal excitability, and firing patterns depend on the astrocytic capacity for buffering K^+^ ions. Extracellular K^+^ concentration changes the equilibrium potential for this ion, thereby affecting the repolarization and hyperpolarization phases of the action potential. In absence of astrocytes, accumulation of extracellular K^+^ during neuronal activity will depolarize the neuronal membrane, inducing tonic spike firing and eventually blocking neuronal activity and conductivity. To prevent these detrimental effects, astrocytes buffer up to 80% of the released K^+^ via Kir4.1 channels [66]. Ki4.1-mediated K^+^ buffering works in concert with astrocytic glutamate transporters and Na+/K+-ATPases [67], thereby maintaining ionic homeostasis in the extracellular space.

In a subpopulation of fast-spiking interneurons, the interstitial brain ECM condensates into peculiar structures known as perineuronal nets [9]. Neuronal activity consolidates the diffusely expressed polyanionic macromolecules into densely packed lattice-shaped layers that can sequester K^+^ ions extracellularly [68, 69]. The reduced synthesis of hyaluronan, which is one of the largest anionic polysaccharides in the extracellular space, causes altered neuronal activity and seizures [8]. Therefore, both astrocytic Kir4.1 channels and polyanionic ECM are critical for K^+^ buffering and neuronal activity maintenance.

### Metabolic regulation of neuronal activity

Neurons are arguably the most energy-demanding cells of the body that do not maintain their own consumption of metabolites. Neuronal metabolism is dominated by mitochondrial oxidative phosphorylation (OXPHOS), which has the highest ATP production efficiency among known metabolic pathways [70]. Glycolysis is strongly downregulated in mature neurons, making them dependent on the import of lactate via the astrocyte-neuron lactate shuttle pathway (Fig. 1). In contrast, astrocytes predominantly use glycolysis that supports the biosynthesis of lipids, nucleotides, and amino acids, and produce ample amounts of lactate [71]. Under normal conditions, astrocytic glycolysis is fueled by glucose uptake from the blood via glucose transporter GLUT1. Astrocytic glycolysis is pivotal for brain function, and, in case of altered glucose supply from the blood (e.g., in stroke), astrocytes can temporarily sustain glycolysis by deriving glucose-6-phosphate (G6P) from glycogen granules in their cytoplasm [72]. At the last step of glycolysis, astrocytes convert most of the produced pyruvate into lactate, which is released into the extracellular space by the monocarboxylate transporters MCT1 and MCT4. Neurons import extracellular lactate using MCT2 and convert it back to pyruvate for fueling the mitochondrial tricarboxylic acid (TCA) cycle and OXPHOS. Thereby, astrocyte-neuron lactate proves the key source of energy for the maintenance of neuronal activity.

In postsynaptic neurons, lactate potentiates NMDAR-mediated currents [73] by increasing NADH/NAD^+^ ratio. NMDAR potentiation by lactate depends on the redox sensitivity of the NR1 subunit [74] and is required for long-term memory formation [75]. Therefore, the transfer of lactate by the astrocyte-neuron lactate shuttle is an essential metabolic mechanism of neuroplasticity.

Neurotransmitters released during neuronal activity provide another crucial substrate for astrocytic metabolism. Following the uptake of glutamate via EAATs, glutamine synthase (GS) catalyzes the condensation of glutamate and ammonia to form glutamine. The importance of this reaction is evidenced by the ample expression of GS in astrocytes. With minor limitations, GS can be considered an astrocytic marker protein [76]. Astrocytic glutamine can be transferred to neurons by several transporter systems (for review, see [77]), of which sodium-coupled neutral amino acid transporters SNAT3 and SNAT5 (also known as SN1 and SN2, correspondingly) localize on the astrocytic membranes, and SNAT1 (also known as SAT1) and SNAT7 are predominantly neuronal [78]. In neurons, glutamine is converted back to glutamate by phosphate-activated glutaminase (PAG), thereby creating the astrocyte-neuronal glutamate-glutamine cycle [79], as shown in Fig. 1. GABA enters the glutamate-glutamine cycle via the TCA cycle through conversion to succinic semialdehyde (SS) and succinate in astrocytic mitochondria [80]. Glutamate is then synthesized from α-ketoglutarate and converted to glutamine by GS. In inhibitory neurons, astrocytic glutamine is one of the main precursors of GABA synthesis [81].

Glutamine transport supports synaptic plasticity by providing the essential substrate for neurotransmitter synthesis in both glutamate- and GABAergic neurons [82]. Genetic disruption of SNAT1 hampered neuronal GABA synthesis and impaired neurotransmission, plasticity, and cortical processing [83] in mice. Inhibition of GS results in reduced synaptic efficacy and altered long-term potentiation (LTP) [84]. Conclusively, the astrocyte-neuronal glutamate-glutamine cycle is critical for neurotransmission and neuroplasticity regulation.

### Remodeling synaptic connectivity in the adult brain

Neuronal networks generate complex activity patterns that require not only the adjustment of synaptic strengths but also the dynamic remodeling of synaptic connectivity. Essential connectivity is established in the juvenile brain before the end of the critical plasticity period [85], which is signified by the maturation of excitatory and inhibitory neuronal networks [86] and the enrichment of plasticity-restricting components of the ECM [87]. Removing and establishing synapses in the mature CNS is mediated by microglia, the immune cells of the brain, and requires their coordinated interaction with astrocytes and ECM.

In the adult brain, both interstitial matrix and perineuronal nets contain axon-repelling chondroitin sulfate glycosaminoglycans (CSPGs) and semaphorins, which restrict new synapse formation [88, 89]. Thereby, CSPG-rich zones such as perineuronal nets compartmentalize neuronal surfaces and create permissive and restrictive microdomains that allow for highly precise targeting of the newly formed synapses. At the same time, ECM integrity is essential for maintaining inhibitory control in neuronal networks, and the depletion of ECM triggers excessive synchronization of neuronal activity [90]. Therefore, retaining excitation-inhibition balance during synaptic reorganization requires restricted ECM degradation that is confined by the area of remodeling. This locality can be achieved through the controlled release of matrix metalloproteases (MMPs) by glial cells [91] and highly accurate synapse elimination by microglia [92, 93].

Microglia are brain-resident macrophages [94] that continually survey the microenvironment [95] and rapidly detect tissue damage [96] or neuronal activity changes [97]. Neuronal activity promotes microglial surveillance via norepinephrine [98] and GABA signaling [99]. Microglial processes establish intercellular junction contacts with neuronal bodies [97]. Within these contacts, microglial purinergic receptors are co-clustered with neuronal potassium channels, creating a specialized nano-architecture optimized for purinergic cell-to-cell communication. Since the activity of a neuron highly correlates with mitochondrial ATP production, the microglia-neuron junctions allow for the rapid stimulation of microglia via ATP/ADP signaling. In stimulated microglia, Ca^2+^ signaling attunes to neuronal activity [100]. Neuronal interleukin 33 (IL33) regulates microglial activity under physiological conditions and promotes the phagocytosis of ECM components by microglia [101]. Neuronal activity regulates microglial phagocytosis [92], but, to the best of our knowledge, evidence for activity-dependent ECM remodeling by microglia is lacking currently. Although further studies are required to decipher the role of microglia-ECM interactions in synaptic remodeling, the available data suggests that microglia can locally remodel ECM, eliminate synapses, and promote synaptic plasticity depending on neuronal activity. Of note, inhibitory synapses are preferentially sculpted by GABA-receptive microglia involving GABA_B_R-mediated signaling [102].

The specificity of activity-dependent synapse elimination is ensured by the phagocytic complement components C1q and C3 in microglia [92] and by exposure of phosphatidyl serine and pentraxins on presynaptic membranes [103, 104]. While the expression of microglial C1q/C3 can be induced by neuronal and astrocytic IL33 release [101, 105], the regulatory mechanisms of presynaptic expression of pentraxins and phosphatidyl serine remain to be understood.

As key mediators of synaptic remodeling, microglia cells contribute to the maintenance of excitation-inhibition balance on the network level. The patrolling function of microglia is regulated by extracellular chemokines including ATP [106, 107] and fractalkine [108, 109], which allow these cells to travel significant distances in the brain parenchyma [95, 110]. Due to the high mobility, a single stimulated microglia cell can potentially remodel multiple synapses and contribute to ranged modulation of excitation and inhibition. Microglia shape the developing neural circuits by engulfing excessive immature synapses via the complement receptor 3(CR3)/C3-dependent phagocytosis [92], a mechanism that is essential for normal synaptogenesis during brain development [111]. Interestingly, a similar mechanism contributes to pathological synapse loss due to upregulated microglial phagocytosis in Alzheimer’s disease [112]. In a healthy brain, microglia can also shape synaptic connectivity using complement-independent mechanisms including trogocytosis [93] and TWEAK signaling [113].

While the possibility of establishing new synapses in mature neuronal networks can be attributed to the local ECM degradation and synapse elimination by microglia, the capability of establishing new synapses is defined by the regulatory molecules produced by astrocytes [114]. Thrombospondin 1 (TSP1) induces the formation of structurally complete but functionally silent synapses. The establishment of active synapses requires the enrollment of glypicans 4 and 6 (GPC4/6) and hevin (also known as secreted protein acidic and rich in cysteine (SPARC) like 1, or SP1). The synaptogenic function of hevin is antagonized by SPARC [115], which is locally synthesized in astrocytic processes [30], allowing for the dynamic control of new synapse formation.

Collectively, the available evidence suggests that synaptic remodeling is likely orchestrated in three steps: local ECM degradation, synapse elimination by microglia, and astrocyte-mediated synapse formation. To support this hypothesis, further research is needed.

### Neuroinflammation and neuroplasticity

The mechanisms of homeostatic brain activity regulation that we reviewed here can be severely altered by inflammatory signaling in multiple neurological diseases. For example, in stroke, neuroinflammation contributes to both damage and remodeling of brain tissue [116]. In both acute and chronic stroke phases, peripheral blood immune cells invade the ischemic brain parenchyma [117, 118], and preclinical studies indicate that immunomodulatory treatments can promote neurological recovery post stroke [119]. After the onset of cerebral ischemia, the release of alarmins [120] and cytokines triggers the pro-inflammatory microglia phenotype [121], which, in turn, can promote the neurotoxic phenotype in reactive astrocytes [122]. A recent study [123] has demonstrated that the neurotoxicity of ischemic astrocytes involves metabolic switching mediated by STAT3 activation. Post-stroke reactive gliosis alters interstitial ECM composition in the brain, which includes the upregulation of short-chain hyaluronan [124] and tenascin-C [125]. As an endogenous ligand of toll-like receptor 4 and inflammatory regulator, tenascin-C regulates morphological alterations in reactive glia [125, 126]. Post-stroke ECM remodeling also involves perineuronal nets [127, 128], and their transient degradation may support neurological recovery.

Neuroinflammation induces similar alterations in reactive glia and ECM in multiple neurodegenerative diseases including Alzheimer’s, Huntington’s, and multiple sclerosis. In Alzheimer’s disease, the release of amyloid-b induces microglial reprogramming and activation [129], which causes synaptic degeneration by complement-mediated microglial phagocytosis [112]. Activated microglia upregulate multiple ECM-degrading enzymes during neuroinflammation [130]. Most likely, the release of extracellular proteases from reactive microglia contributes to the decomposition of perineuronal nets in Alzheimer’s and Huntington’s diseases [131, 132]. Multiple studies using the experimental autoimmune encephalomyelitis (EAE) model of multiple sclerosis demonstrated that the prolonged reactivity in microglia and astrocytes induces neurodegeneration [133, 134]. A recent study demonstrated that ECM composition in EAE altered due to the compromised glycosaminoglycan metabolism [135], showing that multiple sclerosis progression is associated with the reduced expression of 4-sulfated chondroitin sulfates and the increased synthesis of hyaluronic acid.

The lack of an in-depth mechanistic understanding of ECM-glia interactions during neuroinflammation hinders the development of novel therapies for neurodegenerative diseases. However, we believe that the ongoing studies in the field will propose promising translational approaches in the future.

## Concluding remarks

Interactions between neurons, glia, and ECM allow for highly flexible modulation of neuronal plasticity. Ample evidence indicates that neuroplasticity is not restricted to neurons, and, in certain cases, can be dominated by glia. With multiple questions that remain open, future research on glia-mediated neuroplasticity will extend the current understanding of brain activity regulation.
